# Phosphatidic Acid: From Pleiotropic Functions to Neuronal Pathology

**DOI:** 10.3389/fncel.2019.00002

**Published:** 2019-01-23

**Authors:** Emeline Tanguy, Qili Wang, Hervé Moine, Nicolas Vitale

**Affiliations:** ^1^Institut des Neurosciences Cellulaires et Intégratives (INCI), UPR-3212 Centre National de la Recherche Scientifique & Université de Strasbourg, Strasbourg, France; ^2^Institut de Génétique et de Biologie Moléculaire et Cellulaire (IGBMC), CNRS UMR 7104, INSERM U964, Université de Strasbourg, Illkirch-Graffenstaden, France

**Keywords:** lipid signaling, neuron, neuropathology, phosphatidic acid, phospholipase D

## Abstract

Among the cellular lipids, phosphatidic acid (PA) is a peculiar one as it is at the same time a key building block of phospholipid synthesis and a major lipid second messenger conveying signaling information. The latter is thought to largely occur through the ability of PA to recruit and/or activate specific proteins in restricted compartments and within those only at defined submembrane areas. Furthermore, with its cone-shaped geometry PA locally changes membrane topology and may thus be a key player in membrane trafficking events, especially in membrane fusion and fission steps, where lipid remodeling is believed to be crucial. These pleiotropic cellular functions of PA, including phospholipid synthesis and homeostasis together with important signaling activity, imply that perturbations of PA metabolism could lead to serious pathological conditions. In this mini-review article, after outlining the main cellular functions of PA, we highlight the different neurological diseases that could, at least in part, be attributed to an alteration in PA synthesis and/or catabolism.

## Introduction

**Figure 1:**
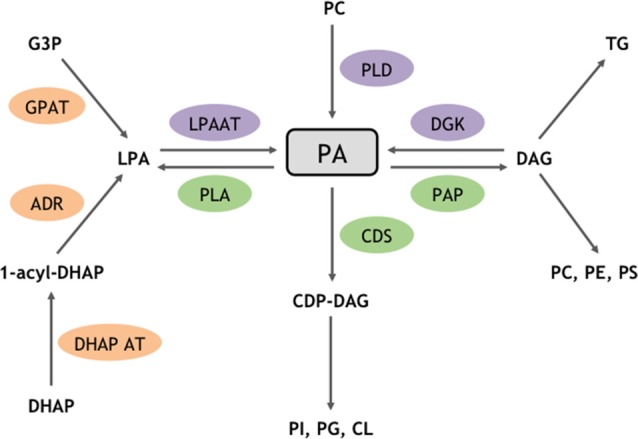
Enzymatic routes for structural and signaling PA metabolism. PA is a major phospholipid for biosynthetic and signaling reactions. Enzymes highlighted in orange are involved in biosynthetic reactions that produce structural PA, whereas enzymes triggering the formation of signaling pools of PA are shown in purple. Enzymes responsible for PA catabolism are shown in green. ADR, Acyldihydroxyacetone-phosphate reductase; CDP, cytidyl phosphatidate; CDS, CDP-diacylglycerol (DAG) synthase; CL, cardiolipin; DHAP, dihydroxyacétone phosphate; DHAP AT, dihydroxyacétone phosphate acyltransferase; G3P, glycerol 3-phosphate; GPAT, glycerol phosphate acyltransferase; PAP, phosphatidic acid phosphatase; PC, phosphatidylcholine; PG, phosphatidylglycerol; PE, phosphatidylethanolamine; PI, phosphatidylinositol; PS, phosphatidylserine; PLA, phospholipase A; TG, triglyceride.

Phosphatidic acid (PA) is a low abundant phospholipid of membranes that, nevertheless, constitutes the original building block from which most glycerophospholipids are synthesized, thus plays an important structural task. Interestingly it was later shown that PA also acts to transmit, amplify, and regulate a great number of intracellular signaling pathways and cellular functions. In cells, PA can be synthesized through different enzymatic pathways (Ammar et al., 2014). Structural PA results from two successive acylation reactions (Figure 1). Signaling PA instead, results from three biosynthesis alternative pathways. The first pathway includes the phosphorylation of diacylglycerol (DAG) by any of the 10 DAG-kinases (DGKs) in mammals (Figure 1). Hydrolysis of the distal phosphodiester bond in phospholipids by phospholipases D (PLD) constitutes the second pathway (Figure 1). Although six different PLDs have been identified in mammals, only PLD1/2 and PLD6 have been shown to synthesize PA from phosphatidylcholine (PC) and cardiolipin (CL), respectively (Jang et al., 2012). The third and final biosynthetic pathway involves acylation of lyso-PA by lyso-PA-acyltransferase (LPAAT) enzymes (Figure 1).

Chemically, PA is composed of a glycerol backbone esterified with two fatty acyl chains at positions C-1 and C-2, and with a phosphate at position C-3. The latter confers the specific features of PA compared to the other diacyl–glycerophospholipids. Indeed, the small anionic phosphate headgroup provides to PA a combination of unique cone-shaped geometry and negative charge (Jenkins and Frohman, 2005). At the molecular level, these two characteristics enable PA both to interact with different enzymes to regulate their catalytic activity and/or their association with membrane compartments and also to affect membrane geometry by creating local negative curvatures (Kooijman et al., 2003). As a consequence, PA has been involved in various important cellular functions including membrane trafficking events where membrane rearrangements are necessary (Bader and Vitale, 2009). In this article, we will present some of the most studied PA identified partners, summarize the most well-described cellular processes that require PA and discuss the potential involvement of an alteration in PA synthesis and/or catabolism in different neurological diseases.

## PA Interacts With and Recruits Numerous Proteins to Membranes

Having an overall view of the interaction network of a given molecule is particularly helpful for deciphering the relationships between the constituents of interactomes and characterizing their function in cell signaling. Since the early description of a handful of proteins that bind to PA, at least using the minimal *in vitro* protein-lipid overlay assay, an extensive list of PA interactors has only emerged more recently (Stace and Ktistakis, 2006). For at least some of those, their interaction with PA appears rather specific with little or no interaction with other negatively charged lipids. The position of PA’s phosphomonoester headgroup in proximity of the interface of acyl chain headgroup was proposed to be important for binding to specific proteins. Supporting the physiological importance of interactions between PA and proteins, numerous proteins have gained domains that display some level of binding specificity for PA (Jang et al., 2012). Although no clear PA-binding domain can be defined at the three dimensional or secondary structural levels, different factors can influence PA interaction with specific domains in target proteins (Tanguy et al., 2018). For instance, we and others have found that some PA-binding modules possess some levels of specificity for the fatty acyl chains of PA (Kassas et al., 2017). In addition, the local membrane environment surrounding PA also appears to modulate PA binding to these modules (Kassas et al., 2017). Finally, it is most likely that PA-binding domains act first through positively charged residues that initially sample for the negative charge of PA buried within the membrane. This first step is probably followed by a docking state where hydrophobic interaction between hydrophobic residues of the module and the fatty acyl chains of PA stabilize the PA-protein interaction (Potocký et al., 2014; Tanguy et al., 2018). At present, more than 50 different proteins have been shown to directly interact with PA, as extensively reviewed in Jang et al. (2012). Briefly, these PA interactors can be classified in four major families.

### Nucleotide-Binding Proteins

Nucleotide (ATP, cAMP, GTP)-binding proteins are important signaling proteins for which the activity is usually regulated by nucleotide binding. Noticeably, the localization and/or activity of many of those proteins are also controlled by PA interaction. This is for instance the case for some of the small GTP-binding proteins of the ADP ribosylation factor (Arf) and Rho (Ras homologous) families that are key players in cytoskeleton remodeling and membrane dynamics. The minimal PA-binding regions of these small GTPases remains however to be defined precisely. Several c-AMP specific phosphodiesterases also interact with PA through their amino-terminal regulatory domains leading to an increase of their enzymatic activity and therefore to a reduction of cAMP levels.

### Regulators of GTP-Binding Proteins

The GTPase-activating proteins ArfGAP with GTPase Domain, Ankyrin Repeat and PH domain 1 (AGAP1) and Regulator of G protein Signaling 4 (RGS4) are negative regulators of Arf and Gα GTP-binding proteins, respectively. Intriguingly, while PA stimulates the GTP-hydrolysis activity of AGAP1, it inhibits that of RGS4, highlighting the multiple and sometimes opposite actions of PA on GTP-binding activity. Furthermore, PA has also been shown to recruit and activate different guanine nucleotide-exchange proteins for small GTPases, such as DedicatOr of CytoKinesis 2 (DOCK2) and Son Of Sevenless (SOS), promoting GTP-binding and activation of Rac and Ras, respectively. It is therefore important to have in mind that PA enrichment in particular sub-membrane domains could influence in different and sometimes contradictory manners a given signaling pathway involving GTP-binding proteins by acting at different stages.

### Kinases

Protein kinases are among the main signaling regulators with nearly 600 different genes. Among those, protein kinase C (PKC) is one of the largest subgroup and PA modulates the activity of several PKC isoforms. PA also promotes recruitment and activation of the proto-oncogene kinase Raf, acting as a gatekeeper in the ERK1/2 pathway. In addition, PA binding to the FKBP12-rapamycin binding region of mTOR is in competition with FKBP12/rapamycin complex of mTOR and is thus likely to influence nutrient sensing and cell proliferation.

Furthermore, lipid kinases contribute to the great diversity of lipids in cells. Among those, PA is capable to stimulate the action of phosphatidylinositol (PI) 4-phosphate 5-kinase (PI4P5K), to promote the synthesis of PI 4,5-bisphosphate (PI(4,5)P_2_), a key signaling lipid (Stace and Ktistakis, 2006; Bader and Vitale, 2009). Finally, cytosolic sphingosine kinase that transforms sphingosine into sphingosine-phosphate appears to translocate to the plasma membrane under the control of PA levels, which most likely affects the signaling pathways involving these two lipids. In conclusion, PA binding modules are found in members of the two major kinase families and consequently, the presence of PA in local membrane composition is expected to influence crucial signaling nodes and the various associated key cellular functions.

### Phosphatases

In addition to kinases, phosphatases constitute the second important family of signaling proteins that modulate protein activity by removing the phosphate residues added by kinases. The protein-tyrosine phosphatase SHP-1 that negatively modulates signaling pathways involving receptor-tyrosine kinase directly interacts with PA therefore triggering phosphatase activity. Furthermore, PA inhibits the enzymatic activity of protein phosphatase 1 (PP1), involved in many cellular activities such as the metabolism of glycogen, the processing of RNA, and the regulation of cell cycle. In conclusion, although not as well described as for kinases, the regulation of several phosphatases by PA offers the possibility of complex and often paradoxical regulation of signaling pathway by a single lipid family.

## The Pleiotropic Cellular Functions of PA

### Actin Cytoskeleton Dynamics

Most cellular functions are influenced by precise cell shapes that are under the control of the cytoskeleton proteins network. Among those the dynamics of the cytoskeleton depends on the formation of actin filaments from a pool of cytosolic monomers, and their subsequent association to each other or to cell membranes, pursued by their depolymerization. Most cellular functions actually depend on a permanent remodeling of this actin network and this is orchestrated in large part by actin binding proteins. Interestingly lipids such as phosphoinositides modulate the affinity of these proteins for actin. PA, however, has also been proposed to participate to this regulation (Ammar et al., 2014).

In neurons, the Rho GTPases and related proteins, through the control of the cytoskeleton, modulate various aspects of cell shape including not only neurite outgrowth and differentiation, axonal growth and targeting, but also dendritic spine formation and maintenance (Ammar et al., 2014). As mentioned in the sections “Nucleotide-Binding Proteins” and “Regulators of GTP-Binding Proteins,” PA synthesized by either PLD or DGK modulates the activity of some different Rho family of GTPases and their regulators by promoting membrane association and/or through the activation of their regulatory proteins (Chae et al., 2008; Nishikimi et al., 2009; Faugaret et al., 2011; Kurooka et al., 2011; Sanematsu et al., 2013). Alternatively, the p21 activated kinases (PAKs) family that regulates various aspects of neuronal development, through actin cytoskeleton reorganization, is also known for being activated by PA (Daniels et al., 1998; Hayashi et al., 2007). Furthermore, PI(4,5)P_2_ is a major lipid regulator of the cytoskeleton and PA is an essential building block leading to PI(4,5)P_2_ synthesis (Figure 1). In an alternative pathway, PA stimulates the phospholipid kinase PI4P5K, leading to the phosphorylation of the membrane phospholipid PI(4)P and the formation of PI(4,5)P_2_ (Honda et al., 1999). In consequence, PA potentially regulates the activity of the three mammalian PI4P5K isozymes that have been described to control actin cytoskeleton reorganization (van den Bout and Divecha, 2009; Roach et al., 2012). Finally, PA levels regulate membrane localization and activity of PKC isoforms α, ε and ζ, all of which are known to affect the morphology of the actin cytoskeleton (Jose Lopez-Andreo et al., 2003).

It is also worth noting that direct interaction of PA with actin-binding proteins has been suggested. Among those potential candidates, the actin-binding protein vinculin known to be involved in neurite outgrowth is a good example (Ito et al., 1982; Johnson and Craig, 1995), but the specificity of these observations remains to be firmly established, since vinculin also binds to other negatively charged lipids, such as PI(4,5)P_2_.

### Membrane Remodeling Events

The secretory pathways have evolved through the establishment of specialized subcellular compartments dedicated to specific biochemical tasks. Membrane trafficking events between these compartments enable particular cells of complex organisms to secrete informative molecules such as hormones, cytokines, and neurotransmitters, for long distance inter-cellular communication. In addition to dedicated and specialized protein machineries, trafficking events of the regulated exocytosis and endocytosis steps also involve remarkable membrane rearrangements that rely on specific lipids (Ammar et al., 2013b). Hence, the first direct molecular data suggesting a role for PLD1-generated PA in hormone release were obtained in chromaffin cells, where overexpression of PLD1, injection of a catalytically-inactive PLD1 mutant (Vitale et al., 2001) or PLD1 silencing (Zeniou-Meyer et al., 2007) affected catecholamine release rates. Using similar approaches, PA synthesized by PLD1 was also shown to govern the regulated secretion of insulin from β-pancreatic cells (Waselle et al., 2005), of von Willebrand factor from endothelial cells (Disse et al., 2009), and acrosomal exocytosis from sperm cells (Lopez et al., 2012; Pelletán et al., 2015). An additional contribution for PA in secretion has been established during the early phase of azurophilic granules release in neutrophils triggered by anti-neutrophil cytoplasmic antibodies (Williams et al., 2007). Ultimately, different enzymes controlling PA metabolism such as PLDs, LPAATs and DGKs have been proposed to regulate neurotransmission in several neuronal models, suggesting that PA regulates synaptic vesicle release and cycle (Humeau et al., 2001; Schwarz et al., 2011; Tabet et al., 2016a,b; Raben and Barber, 2017).

Intense membrane remodeling also occurs in specialized phagocytic cells, such as in macrophages. Indeed, the ingestion of pathogens, cell debris, or any other solid particle through the formation of phagosomes requires plasma membrane extension and either local lipid synthesis, transfer, or vesicular fusion. PA synthesis by PLD2 has been shown to be important for this early step of phagocytosis, while PA synthesis by PLD1 appears to be also important for the later step of phagosome maturation (Corrotte et al., 2006, 2010). Of note, PA transfer from the ER to plasma membrane during “frustrated phagocytosis,” a model where macrophages are plated on IgG-coated plates, has also been suggested to occur from experiments using a combination of lipidomic analysis performed on subcellular fractions and novel PA sensors (Kassas et al., 2017). Furthermore, PA is involved in the invasion and exit of infected cells by apicomplexan parasites (Bullen et al., 2016). It was shown that PA is required for the release of adhesins, perforins and proteases from specialized organelles from these parasites called micronemes (Bullen et al., 2016).

**Figure 2:**
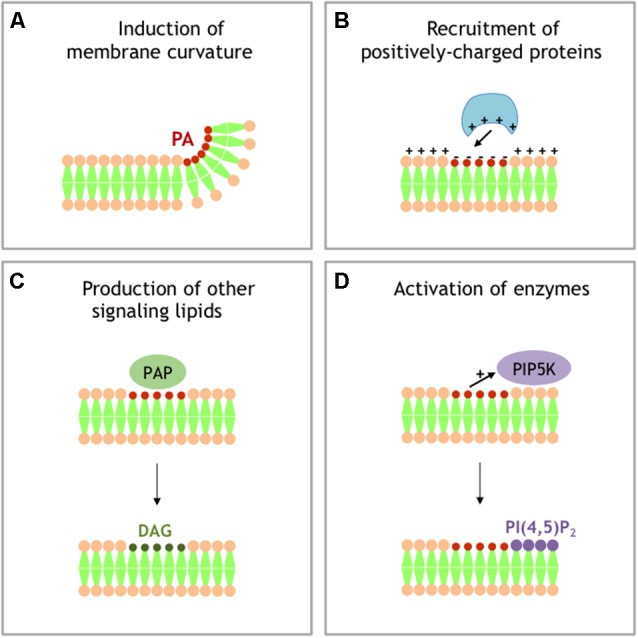
Different models for signaling activity of PA.** (A)** Local accumulation of PA in a single leaflet of membrane generates negative membrane curvature. **(B)** Local accumulation of PA generates local buildup of negative charges that recruit PA effector-containing positively charged domains. **(C)** PAPs transforms PA into DAG, another signaling lipid with specific activity. **(D)** PA stimulates the activity of phosphatidylinositol 4-phosphate 5-kinase (PI4P5K), producing PI 4,5-bisphosphate (PI(4,5)P_2_), an additional important signaling lipid.

Additional intracellular trafficking events involving intense membrane remodeling have also been shown to require modification in PA levels. To cite only a few, we can also mention that PA critically regulates vesicle budding from the Golgi (Yang et al., 2008), autophagy (Holland et al., 2016), and exosome release (Ghossoub et al., 2014). The mechanisms by which PA promotes membrane rearrangements remain however an unsolved issue (Figure 2). The first proposed mode of action of PA in membrane remodeling may depends on its ability to induce membrane curvature and promote fusion, but its ability to specifically regulate the activity of different proteins involved in the vesicle docking and/or recruit crucial fusion proteins has also been proposed (Tanguy et al., 2016, 2018). In a simplified model for membrane fusion a mixture of lipids and proteins appear to be crucial at the fusion site (Tanguy et al., 2016). In principle the intrinsic negative curvatures of accumulating cone-shaped lipids, such as PA in the inner (cis) leaflets of contacting bilayers, should facilitate fusion of merging membranes. But it is worth noting that the promotion of membrane fusion through local modification of membrane curvature also appears to hold true for other cone-shaped lipids such as cholesterol and DAG (Tanguy et al., 2016). Reconstituted membrane fusion assays have been valuable to dissect the role of individual components and on this instance it is important to highlight that PA was observed playing a unique role among cone-shaped lipids in a yeast vacuole fusion assay, suggesting a more complex mode of action of this lipid (Mima and Wickner, 2009). Furthermore, PA could locally accumulate and form microdomains highly negatively charged, which potentially serve as membrane retention sites for several proteins key for exocytosis, such as the SNARE protein syntaxin-1 (Lam et al., 2008), or other membrane remodeling processes (Jenkins and Frohman, 2005). Finally, as a precursor for DAG and PI(4,5)P_2_, both known to contribute to numerous membrane remodeling events, PA could also have indirect effects. All these potential contributions of PA in membrane fusion have been reviewed elsewhere (Chasserot-Golaz et al., 2010; Ammar et al., 2013b, 2014; Tanguy et al., 2016), but solving the issue of the mechanistic role of PA in a given membrane remodeling process requires probing these different scenarios (Figure 2), which is now in need for novel methods and tools.

### Apoptosis, Survival, Growth, Proliferation and Migration

Many survival signals including hormones and growth factors activate PA synthesis through the stimulation of PLD activity. Similarly, mitogenic signals trigger cell proliferation, suppression of cell cycle arrest, and prevention of apoptosis. The PLD–PA–Rheb–mTOR and the PLD–PA–MAP kinase pathways are the two main downstream pathways of PLD involved in mitogenic signals and have been described extensively (Foster, 2009). Obviously, future solving of the complex imbrication of these pathways and understanding of the spatiotemporal relationships between PA-generating enzymes, PA-binding partners and PA itself will require development of more specific tools and extensive work.

## Neurological Disorders Potentially Linked to an Alteration of PA Levels

In all organisms from yeast to mammals, PA was shown to possess signaling activity (Jenkins and Frohman, 2005) and a recent review highlights the apparent mystery of the many roles of PA in plants (Pokotylo et al., 2018). In addition, various PA-generating enzymes were shown to be involved in an increasing number of neuronal pathologies, suggesting a fundamental role of PA in the outcome of these neurodiseases (Tanguy et al., 2018). In the next chapter, we will describe four neuronal pathologies that may be the consequence, at least partially, of an alteration in PA dynamics.

### Fetal Alcohol Spectrum Disorders

The damaging effects of alcohol drinking during gestation on the developing fetus are extremely well documented (Ehrhart et al., 2018). Fetal alcohol spectrum disorders (FASDs) is a generic term used to define the birth deficiencies that result from prenatal exposure to alcohol that range from mild to severe. These developmental defects on unborn infants have lifelong physical, behavioral, and cognitive disabilities. As alcohol consumption avoidance during pregnancy is in theory easy to achieve, FASD is in fact considered as one of the largest preventable forms of non-genetic birth disabilities associated with intellectual incapacity.

Although the main effort remains prevention of alcohol consumption during pregnancy, it is also important to understand the underlying pathological mechanisms involved in these effects of ethanol. In addition to the well-recognized ethanol and acetaldehyde toxicity, alcohol intensifies oxidative stress causing consequent effects such as DNA, protein and membrane damages. Additionally, it has been known for over three decades that in the presence of 1%–3% of ethanol, the two best characterized mammalian isoforms PLD1 and PLD2 catalyze a transphosphatidylation reaction. In this case alcohol replaces water during PC hydrolysis, and phosphatidyl alcohols are formed at the expense of PA (Jenkins and Frohman, 2005). Thus, in presence of ethanol, phosphatidylethanol is synthesized while PA is not. Since it is likely that phosphatidylethanol cannot replace PA as a signaling molecule, this was used as a trick to “inhibit” PLD activity (actually prevent PA production), but also in assays to quantify PLD activity (Ammar et al., 2014). It was also shown that ethanol inhibits the mitogenic downstream actions of PA on neuron progenitors (Klein, 2005). Furthermore, it was recently shown that both PLD1 and PLD2 strongly contribute to astroglial proliferation induced by IGF-1 (Burkhardt et al., 2014). Therefore, the perturbation of the IGF1-PLD signaling pathway could, at least in part, explain the teratogenic effects of ethanol observed in FASDs.

### Neurological Cancers

Glioblastoma is the most frequent and aggressive brain cancer, with an estimated incidence of near five novel cases per 100,000 persons every year in the USA and Europe. Nearly 200,000 persons die from glioblastoma every year worldwide. It is a relatively difficult cancer to diagnose, as the symptoms are mainly non-specific, including headache and nausea, but leading to alterations of neurological functions such as speech, vision, behavior and memory. Like for many cancer tissues, elevated PLD activity was found in glioblastoma, suggesting that an increase in PA levels is a cause and/or consequence of the pathology (Park et al., 2009). At least part of the survival effect of increased PLD activity on glioblastoma appears to involve the Akt pathway (Bruntz et al., 2014). Interestingly, lipidomic analyses revealed that PA levels are altered in the regions that attract glioblastoma cells, indicating that PA levels control the homing process of glioblastoma (Wildburger et al., 2015). Undoubtedly, a better understanding of the multiple functions of PA in brain tumor development and progression may help to improve treatments and subsequently get a better prognosis for this aggressive cancer.

### Intellectual Disability Diseases

Intellectual disability diseases are a common state defined by significant restriction in intellectual capacities and adaptive behavior that happen during childhood, with an overall intelligence quotient bellow 70 together with associated reduction in social, daily living and communication skills. These heterogeneous disease conditions affect 1%–3% of all populations and are thought to result from multiple causes, including environmental, chromosomal and monogenetic alterations. Among the several hundreds of genes involved, some affect brain development, neurogenesis, neuronal migration, or synaptic function (Humeau et al., 2009). Below we will briefly describe the data that support the notion of an alteration of PA levels and/or dynamics in the Fragile-X syndrome (FXS) and the Coffin-Lowry syndrome (CLS).

FXS is a neurodevelopmental pathology accountable for the most common inherited form of intellectual infirmity and autism spectrum disorder. It is generally the consequence of the hypermethylation of CGG expansion repeats (>200) in the 5′ untranslated region of the *FMR1* gene leading to transcription silencing. In a recent study, we pointed DGK kinase-κ (DGKκ) mRNA as the foremost target of Fragile Mental Retardation Protein and found an alteration in PA synthesis in neurons cultured from *Fmr1*-knockout mice after group 1 metabotropic glutamate receptor (mGluRI) stimulation (Tabet et al., 2016a). Silencing DGKκ in CA1 pyramidal neurons modified the immature over mature spine ratio and like in the *Fmr1*-knockout mouse phenotype, reduced LTP and increased LTD (Tabet et al., 2016a). Moreover, the typical *Fmr1*-knockout mouse phenotype on dendritic spine morphology was restored back to normal after overexpression of DGKκ (Tabet et al., 2016a). Finally, DGKκ silencing by shRNA in the mouse reiterated autistic behaviors, such as impaired social interaction, hyperactivity and altered nest-building very much like those seen in the *Fmr1*-knockout mouse model (Tabet et al., 2016a). Based on these observations, it was proposed that a major molecular consequence of the loss of FMRP expression in FXS is to prevent DGKκ translation, leading to an alteration in DAG and PA levels in neurons (Tabet et al., 2016b). A main consequence of this imbalance would be the alteration of the downstream signaling of DAG and PA required for maturation of dendritic spines and establishment of correct synaptic plasticity (Moine and Vitale, 2018).

Loss of function mutations in the gene encoding Ribosomal S6 Kinase 2 (RSK2) lead to CLS, a rare syndromic form of mental retardation that shows X-linked inheritance. However, the molecular bases of the major neuronal alterations of CLS, such as moderate to severe defect in neurodevelopment, remain indefinable. In agreement with the notion that PLD1-generated PA is key to neurite outgrowth, we observed significant delayed in *Pld1* knockout neuron maturation (Ammar et al., 2013a). These observations were as well found in a mouse model for CLS syndrome since *Rsk2* knockout neurons exhibited developmental delay (Ammar et al., 2013a). Furthermore, RSK2 phosphorylates PLD1 at threonine 147 when exocytosis was triggered (Zeniou-Meyer et al., 2008) or during neurite outgrowth (Ammar et al., 2013a) in PC12 cells. A specific sensor for PA revealed an increase in PA levels at the tips of growing neurites in neurons resulting from PLD1 activation (Ammar et al., 2013a). Interestingly, PLD1 was found to be associated with BDNF positive endosomes (Ammar et al., 2015) and with vesicular structures derived from the trans Golgi, co-labeled by the vesicular SNARE VAMP-7/TiVAMP (Ammar et al., 2013a). The fusion efficiency of these PLD1/VAMP-7 vesicles in the growth cone was severely impaired by RSK2 and PLD1 inhibitors, suggesting that both PLD1 and RSK2 are necessary for membrane provision needed during neurite outgrowth (Ammar et al., 2013a). Accordingly, co-immunoprecipitation and confocal colocalization experiments indicated that RSK2 and PLD1 are found in a complex at the tip end of growing neurites, supporting the observation of an increased PA level at this location (Ammar et al., 2013a). Altogether, these results have highlighted the importance of PA-mediated membrane trafficking in neurite outgrowth and a key role of RSK2 in PA synthesis during this process, by phosphorylation and subsequent activation of PLD1. In consequence, it has been proposed that at least some of the clinical consequences of the CLS might result from an inadequate PA production during neuronal development and function (Zeniou-Meyer et al., 2010).

### Neurodegeneration

It is becoming more and more evident that human neurodegenerative diseases such as Alzheimer disease (AD) also have a critical lipidic feature in their outcome. This aspect has been first pointed out by the susceptibility of the ApoE4 allele to AD, but more recently PLDs have also been proposed to contribute to the development of the pathology. It was first shown that PLD1 is involved in the vesicular trafficking of βAPP (Cai et al., 2006) and later that increased expression of APP promoted PLD activity in human astroglioma cells (Jin et al., 2007). Although highly debated, the observation that a rare variant of PLD3 gene confers susceptibility to AD has put PLD and PA on the spotlight (Cruchaga et al., 2014; Heilmann et al., 2015; Lambert et al., 2015; van der Lee et al., 2015). The most compelling evidence that defects in PA production by PLDs are involved in AD comes from an elegant study combining detailed lipidomics with behavioral tests in mouse models. In this study, the authors found that PLD2 knockout fully rescued AD-related synaptic dysfunction and cognitive deficits in a model of AD (Oliveira et al., 2010). The exact nature of the PA imbalance in AD awaits however to be fully defined and the possibility to interfere with AD condition by correcting this imbalance is probably very far from reach.

## Conclusion

The diversity of mechanisms of PA signaling and physiological functions mostly relies on the fact that PA is synthesized by a complex set of different enzymes involved in diverse array of pathways. PLDs, DGKs, and LPAATs each constitute a big collection of isoenzymes differently localized within cells and displaying cell type specificity. In fact, the specific subcellular distribution, regulation, and/or substrate preferences of these enzymes probably account for the heterogeneity of PA composition in membranes. These aspects, altogether with the capacity of PA-binding modules in proteins to sense the local membrane environment and the type of PA species, offer a hub for the functional diversity of PA from molecular and cellular to physiological functions. There is no doubt that advanced lipidomics in combination with novel imaging tools to follow PA’s dynamics will help to gain a better understanding of the apparent paradox of the abundance of function of this simple lipid. Further understanding of the biophysical side of PA’s action on membranes is also critically needed to provide novel ideas for the treatment of the growing number of neuronal pathologies linked to the alterations of PA metabolism.

## Author Contributions

All authors listed have made a substantial, direct and intellectual contribution to the work, and approved it for publication.

## Conflict of Interest Statement

The authors declare that the research was conducted in the absence of any commercial or financial relationships that could be construed as a potential conflict of interest.
